# Fiber laser technologies for photoacoustic microscopy

**DOI:** 10.1186/s42492-021-00076-y

**Published:** 2021-04-30

**Authors:** Long Jin, Yizhi Liang

**Affiliations:** Guangdong Provincial Key Laboratory of Fiber Optic Sensing and Communications, Institute of Photonics Technology, Jinan University, Guangzhou, 510632 China

**Keywords:** Fiber lasers, Fiber sensors, Photoacoustic imaging, Photoacoustic microscopy

## Abstract

Fiber laser technology has experienced a rapid growth over the past decade owing to increased applications in precision measurement and optical testing, medical care, and industrial applications, including laser welding, cleaning, and manufacturing. A fiber laser can output laser pulses with high energy, a high repetition rate, a controllable wavelength, low noise, and good beam quality, making it applicable in photoacoustic imaging. Herein, recent developments in fiber-laser-based photoacoustic microscopy (PAM) are reviewed. Multispectral PAM can be used to image oxygen saturation or lipid-rich biological tissues by applying a Q-switched fiber laser, a stimulated Raman scattering-based laser source, or a fiber-based supercontinuum source for photoacoustic excitation. PAM can also incorporate a single-mode fiber laser cavity as a high-sensitivity ultrasound sensor by measuring the acoustically induced lasing-frequency shift. Because of their small size and high flexibility, compact head-mounted, wearable, or hand-held imaging modalities and better photoacoustic endoscopes can be enabled using fiber-laser-based PAM.

## Introduction

A fiber laser is a laser whose active gain medium is an optical fiber doped with rare-earth elements, including erbium, ytterbium, and neodymium [1–4]. In general, fiber nonlinearities, such as stimulated Raman scattering (SRS) and stimulated Brillouin scattering, or four-wave mixing, can also provide gain and thus serve as a gain medium for a fiber laser. In the 1980s, erbium-doped fibers were designed and fabricated for the optical amplification of long-haul lightwave communication systems. Equipped with two reflective mirrors, a fiber laser was formed. Continuous-wave and pulsed fiber lasers, including mode-locked and Q-switched lasers, have been studied for fundamental physical research, communications, and industrial applications. High-power fiber lasers have gained significant interest in recent decades and have been widely used in cutting, engraving, welding, and cleaning in the advanced manufacturing industry, taking advantage of the higher output powers or pulse energies, lightweight systems, excellent beam quality, and smaller focus diameters [5–7]. In addition, mode-locked fiber lasers have been applied as optical frequency comb sources in precision measurements and spectroscopy [8]. In biophotonics, fiber lasers serve as an excellent light source for two- or multiphoton imaging, which can map real-time neural activities [9, 10]. A high-power fiber laser, for example, a Tm- or Ho-doped fiber laser working at 2 μm, is also a good candidate for minimally invasive microsurgery [11]. Fiber lasers have also improved the development of sensor technology [12–14]. A short-cavity fiber laser, known as a distributed feedback (DFB) or distributed Bragg reflector laser, can be used as a sensor to detect ultraweak signals, including strains and acoustic waves. Fiber laser sensors can be wavelength-division multiplexed to form a sensor array, which has been applied in seismic monitoring, resource exploration, and defense applications.

**Fig. 1:**
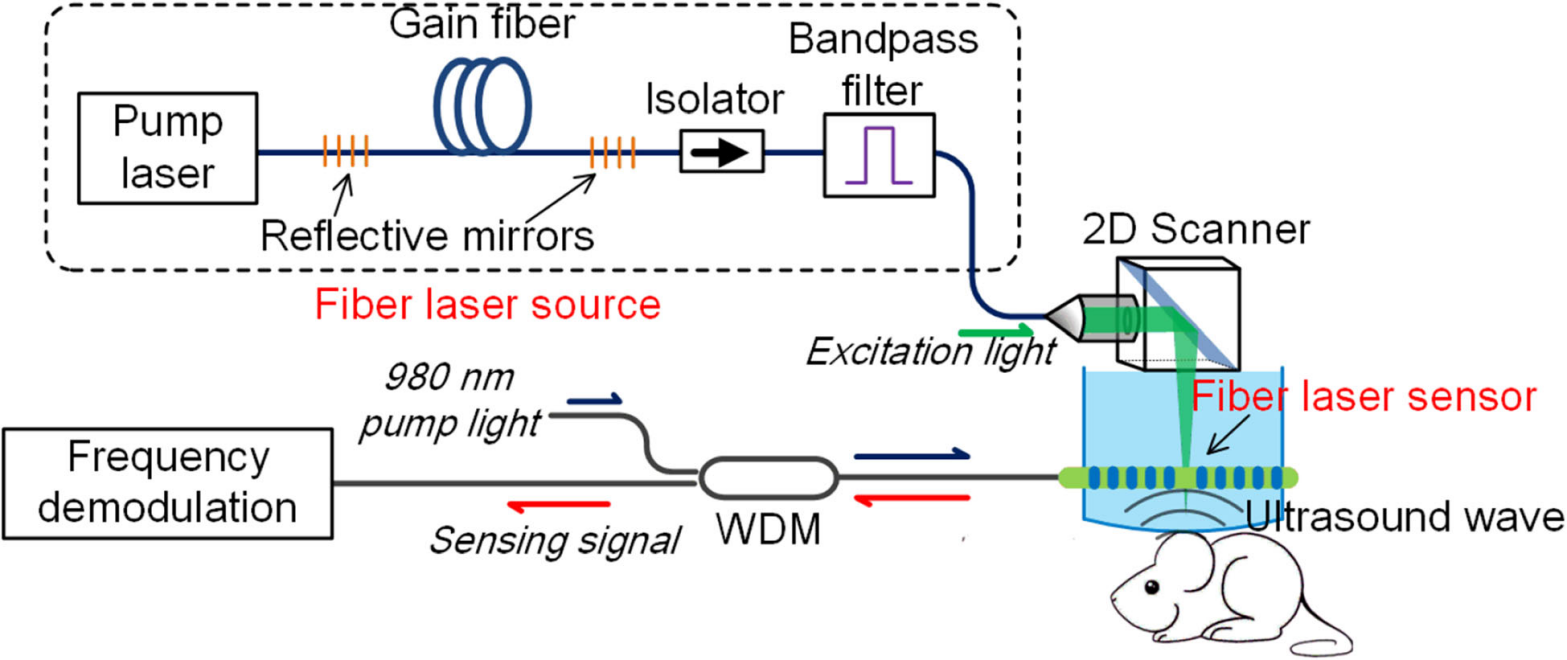
Schematic of fiber-laser-based PAM. Multiwavelength, high-repetition-rate pulsed fiber lasers are used for photoacoustic excitation. A distributed-Bragg-reflector fiber laser is used to detect optically induced ultrasound waves

Photoacoustic imaging (PAI) is a fast-growing technology for life science research and medical diagnostics [15, 16]. As a fundamental form of PAI, photoacoustic microscopy (PAM) is used to image optical absorption in biological tissue at a subcellular resolution by detecting the ultrasound waves induced through optical absorption [17]. Typically, PAM uses a focused pulsed laser beam for photoacoustic excitation and a piezoelectric transducer to detect ultrasound waves to generate an A-line. Two- or three-dimensional imaging can be achieved through mechanical scanning of the probe. A full image consists of multiple A-lines. Here, the development of PAM, which uses fiber lasers for both photoacoustic excitation and ultrasound detection, is reviewed. Figure 1 shows a schematic of a fiber-laser-based PAM. Here, a fiber laser with a high repetition rate, typically tens or hundreds of kilohertz, is used for photoacoustic excitation. It can have an output at the desired wavelength to image different endogenous absorbers (hemoglobin, water, and lipids) and doped contrast agents. In this microscopy, optically induced ultrasound waves are detected by a fiber laser sensor, which translates the pressure waves into a variation within the lasing frequency. The frequency change is read out from the beating signal between two orthogonal lasing modes. This sensor can enable the use of head-mounted or hand-held PAMs, as well as smaller photoacoustic endoscopes.

This review is organized as follows: Fiber laser sources for PAM section describes three types of fiber-based laser sources. Q-switched fiber lasers have been used for many years, and their output wavelength can be tuned within the gain spectrum of rare-earth-doped fibers. In addition, supercontinuum laser sources can continuously cover a spectral range from ultraviolet (UV) to near-infrared and can be employed in PAM/optical coherent tomography (OCT) dual-mode imaging. Finally, SRS based fiber lasers can achieved a multiwavelength output with a quick wavelength switching capability, which is favorable for functional and Gruneisen relaxation PAMs. Fiber-laser-based ultrasound sensors section reviews the working principle, as well as the performance of a fiber laser ultrasound sensor and its application in PAM. Additional applications taking advantage of their small size and flexible geometry are also discussed. Finally, in Discussion and conclusion section, how fiber lasers may revolutionize PA imaging technology to enable better microscopes and endoscopes is described.

## Fiber laser sources for PAM

### Q-switched pulsed fiber lasers

**Fig. 2:**
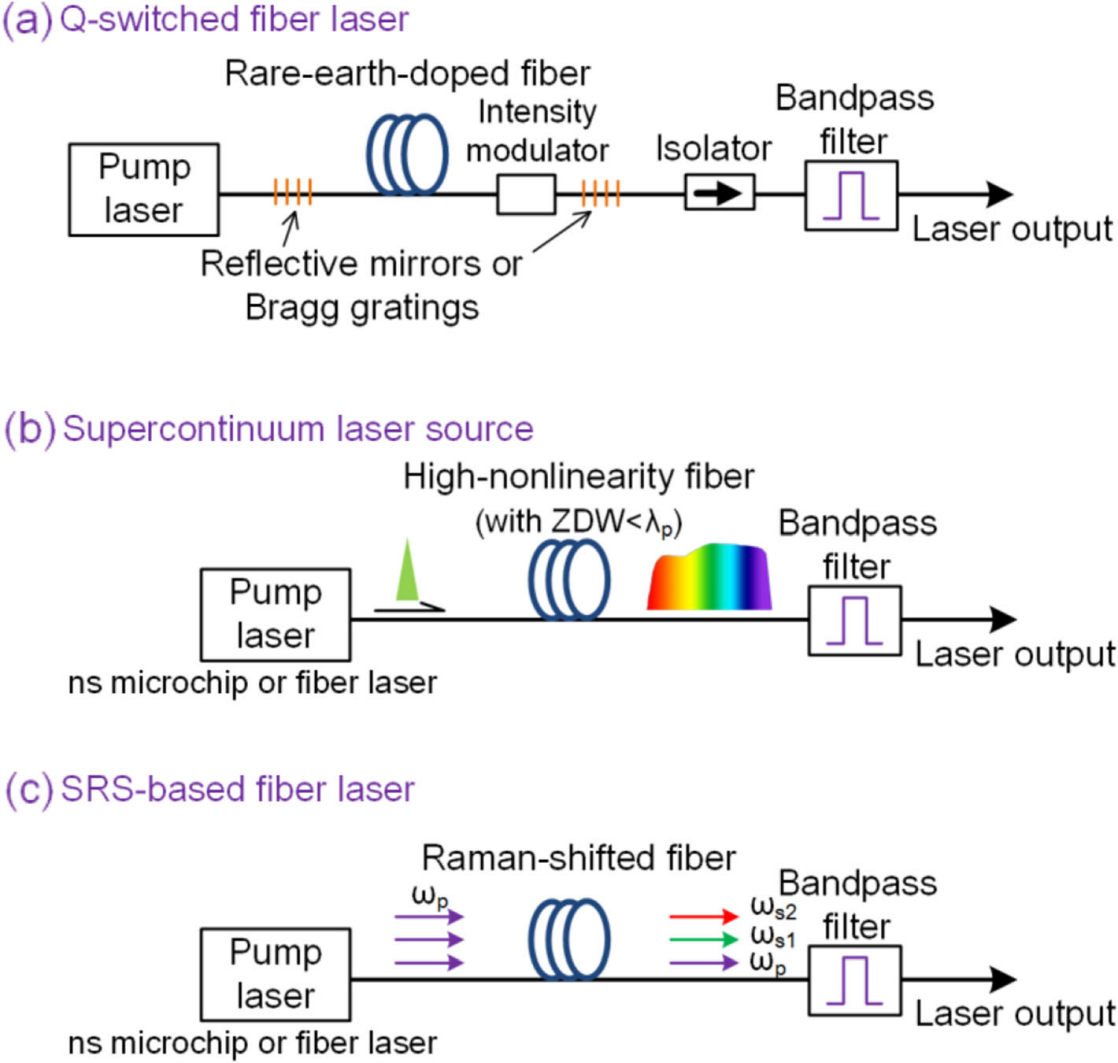
Schematics of fiber laser sources for PAM. (**a**): Q-switched fiber laser; (**b**): Supercontinuum laser; and (**c**): SRS-based fiber laser

Figure 2(a) shows the typical configuration of a Q-switched nanosecond fiber laser. The length of the rare-earth-doped fiber is pumped using a semiconductor laser through a wavelength-division multiplexer to achieve an optical gain. Optical feedback is provided using two reflective mirrors or fiber Bragg gratings with optimal reflectivities. An optical isolator is used to ensure a unidirectional operation. A fused fiber coupler acts as an output coupler with an optimal coupling ratio to maximize the output power. An intensity modulator is applied to periodically modulate the loss of the cavity and generate nanosecond laser pulses. The Yb-doped fiber laser operating at 1064 nm is a cost-effective laser source owing to its high-power output and has found wide applications in advanced manufacturing. A small 1064-nm pulsed fiber laser system with a repetition rate of 50 kHz was used during PAM to image melanoma cells [18]. To image hemoglobin, the laser was frequency-doubled using a potassium titanyl phosphate (KTP) crystal at 532 nm [19].

**Fig. 3:**
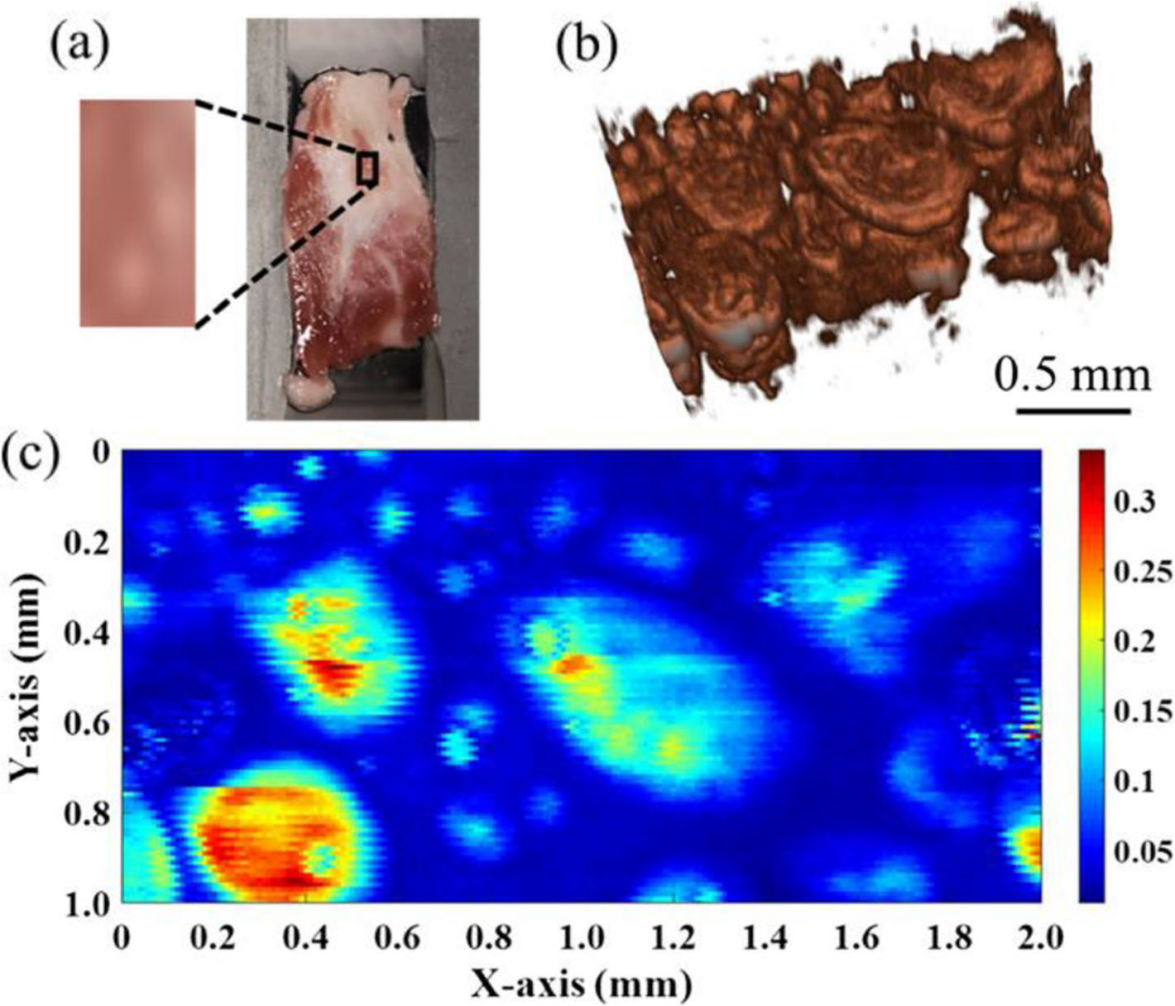
Lipid PAM result with a gain-switched thulium-doped fiber laser at approximately 1720 nm. (**a**): Photograph of a fat beef slice to be imaged; (**b**): 3D imaging; and (**c**): MAP imaging results when scanning the box section in (**a**) [21]

A gain-switched 1.7-μm Tm-doped fiber laser was recently demonstrated for the imaging of lipid-rich tissues when applying PAM [20]. Here, the optical gain was periodically switched by modulating the pump power at 1.55 μm. A Tm-doped fiber, which is typically used for amplification and lasing at 1.9 or 2 μm, can also offer optical gain at 1.7 μm by optimizing the cavity structure. A tunable optical filter was inserted into the cavity to enable a wavelength tuning range of 1690–1765 nm, covering the entire 1720-nm lipid-absorptive band. Stable laser pulses with a duration of 190 ns, pulse energy of 2.2 μJ, and repetition rate of 300 kHz were achieved. The performance of the 1.7-μm fiber laser was further improved with a Bragg-grating-based linear cavity and a much shorter gain fiber length [21]. As a result, the pulse width was reduced to less than 16.7 ns, and the pulse energy was enhanced by a factor of 30 on average for a more efficient photoacoustic excitation. As a result, the imaging quality can be improved, as shown in Fig. 3. By additively introducing a delay path, Gruneisen-relaxation PAM was achieved, which can provide a better spatial resolution [22].

### Supercontinuum light sources

Figure 2(b) shows the configuration of the supercontinuum fiber laser source. Supercontinuum refers to the physical phenomenon of severe spectral broadening of the original pump laser beam as a result of a collection of nonlinear processes. The supercontinuum was first observed in the 1960s in research on Raman spectroscopy and has received significant attention in fundamental scientific research and applications. Supercontinuum generation occurs in a highly nonlinear optical medium with an anomalous dispersion at the pump wavelength. The generation efficiency is extremely low in bulk materials and can be boosted using a photonic crystal fiber (PCF) [23, 24]. A PCF is a pure silica optical fiber consisting of a two-dimensional periodic air hole array in its transverse geometry. By introducing a central defect core, light can be confined with a low transmission loss, based on a modified total reflection mechanism. The optical properties, including the nonlinearity coefficient and dispersion curve, can be engineered by changing the geometrical parameters, such as the air-hole size and lattice period. Such a PCF can have a zero-dispersion wavelength (ZDW) below the pump wavelength and an extended anomalous dispersion range, yielding a broad and flat output spectrum. Currently, a commercial supercontinuum source covers the spectral range from the UV or visible bands to near-infrared bands (typically 450–2000 nm pumped at 1064 nm). PCF-based supercontinuum sources with fs, ps, ns, or even continuous-wave outputs have found applications in frequency metrology, optical coherence tomography, fluorescence lifetime imaging, and gas sensing.

**Fig. 4:**
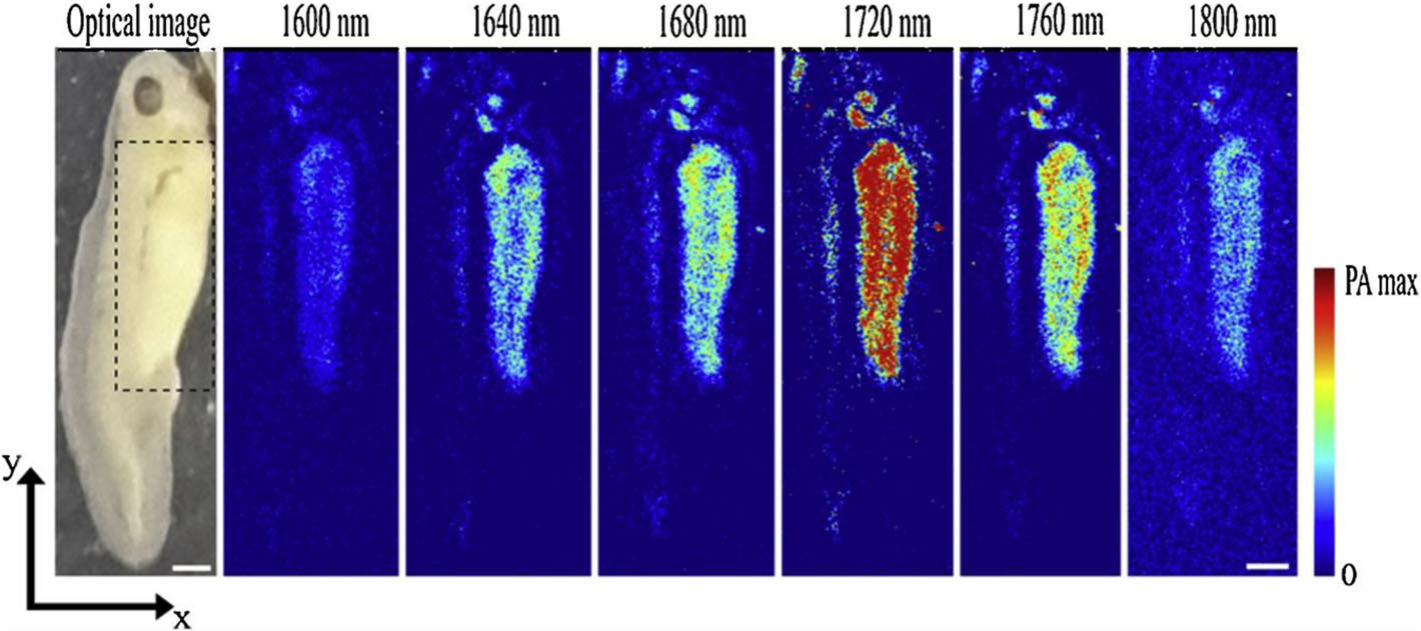
Optical image and six in vivo PAM images of a *Xenopus laevis* tadpole acquired from 1600 to 1800 nm in steps of 40 nm. The highlighted region in the optical image shows the yolk sac. The scale bar represents 1 mm [29]

The idea of using a supercontinuum source for PAM was proposed for multispectral imaging or the detection of lipid-rich tissues, whose absorption band (at 1720 nm) cannot be covered using conventional laser sources. Individual bandpass filters are then applied to filter out the desired spectral components for multispectral photoacoustic excitation [25]. The use of a supercontinuum also makes it convenient to conduct OCT/PAM dual-mode imaging, which offers both absorption and scattering information with a single imaging modality [26]. However, spectral broadening also leads to a low spectral density, which is insufficient for the imaging of biological tissues. Furthermore, by enhancing the power handling capability by using a fiber taper and employing a broader spectral range (500 to 800 nm) for photoacoustic excitation, such a dual-mode modality was used for imaging a living ear [27]. A practical strategy for addressing this problem is to control the broadened spectrum within a bandwidth of less than 200 nm to enhance the spectral density [28]. For example, a near-infrared supercontinuum laser was demonstrated with pumping at 1.55 μm to yield a usable spectral range of 1.65–1.85 μm. Here, a conventional communication optical fiber is employed as the nonlinearity medium, considering the ZDW located at 1.3 μm, which allows significant spectral broadening within this band. The pulse energy density reached approximately 25 nJ/nm at approximately 1750 nm, which was used for the PAM of lipid-rich tissues. Recently, the pulse energy was further enhanced using a directly modulated diode as the pump light [29]. The intensity of the pump light was boosted using multi-stage Erbium and Erbium/Ytterbium codoped fiber amplifiers, followed by a few meters of a dispersion-shifted fiber. This supercontinuum source can cover a band of 1600–1800 nm, with an output energy of hundreds of nanojoules at each wavelength. Figure 4 shows the multispectral, in vivo PAM results of the lipid imaging of a tadpole, scanned using multiple wavelengths of the supercontinuum source. The results show that the photoacoustic signals reach the maximum amplitudes at 1720 nm, which is in agreement with the absorption spectrum of the C-H bond.

### SRS-based fiber laser sources

SRS is a fundamental nonlinear process used in an optical fiber that induces an energy transfer between two different wavelengths and enables broadband optical amplifiers. Figure 2(c) shows a typical schematic of a Raman fiber laser source. When a high-intensity pump laser at *ω*_p_ is injected into an optical fiber, it experiences inelastic scattering into a Stokes wave at *ω*_s_ through an optical phonon with an angular frequency of *ω*_v_ = *ω*_p_ − *ω*_s_. This process is known as Raman scattering. In SRS, the energy of the pump light is continuously transferred to the Stokes wave (which is viewed as a ‘signal’ light) because its frequency difference naturally matches the molecular vibrational frequency. As a result, the Stokes wave is significantly amplified. With a sufficiently high pump intensity, such an amplification process can be ‘duplicated’ to generate multiple higher-order Stokes waves. The *i*th Stokes wave can be viewed as the pump light for the amplification of the (*i +* 1)th Stokes wave. Therefore, SRS is a useful optical nonlinearity for obtaining a laser with new wavelengths by merely pumping an optical fiber with an intense laser [30].

As a preliminary demonstration, a 1064-nm microchip laser was first frequency-doubled to achieve a 532-nm pump laser and then injected into a polarization-maintaining single-mode fiber to achieve an 80-nJ pulse energy at four discrete wavelengths [31]. The wavelength range was extended to 604 nm (the fifth-order Stokes wavelength), and the pulse energy was enhanced to hundreds of nanojoules using a PCF as the Raman medium. The utilization of a pure silica PCF can effectively avoid irreversible damage to the fiber induced through the photodarkening effect [32]. The excitation wavelength was further extended to 810 nm with a pure silica core fiber with high nonlinearity, and in vivo imaging was demonstrated using an optical-resolution PAM scheme [33].

**Fig. 5:**
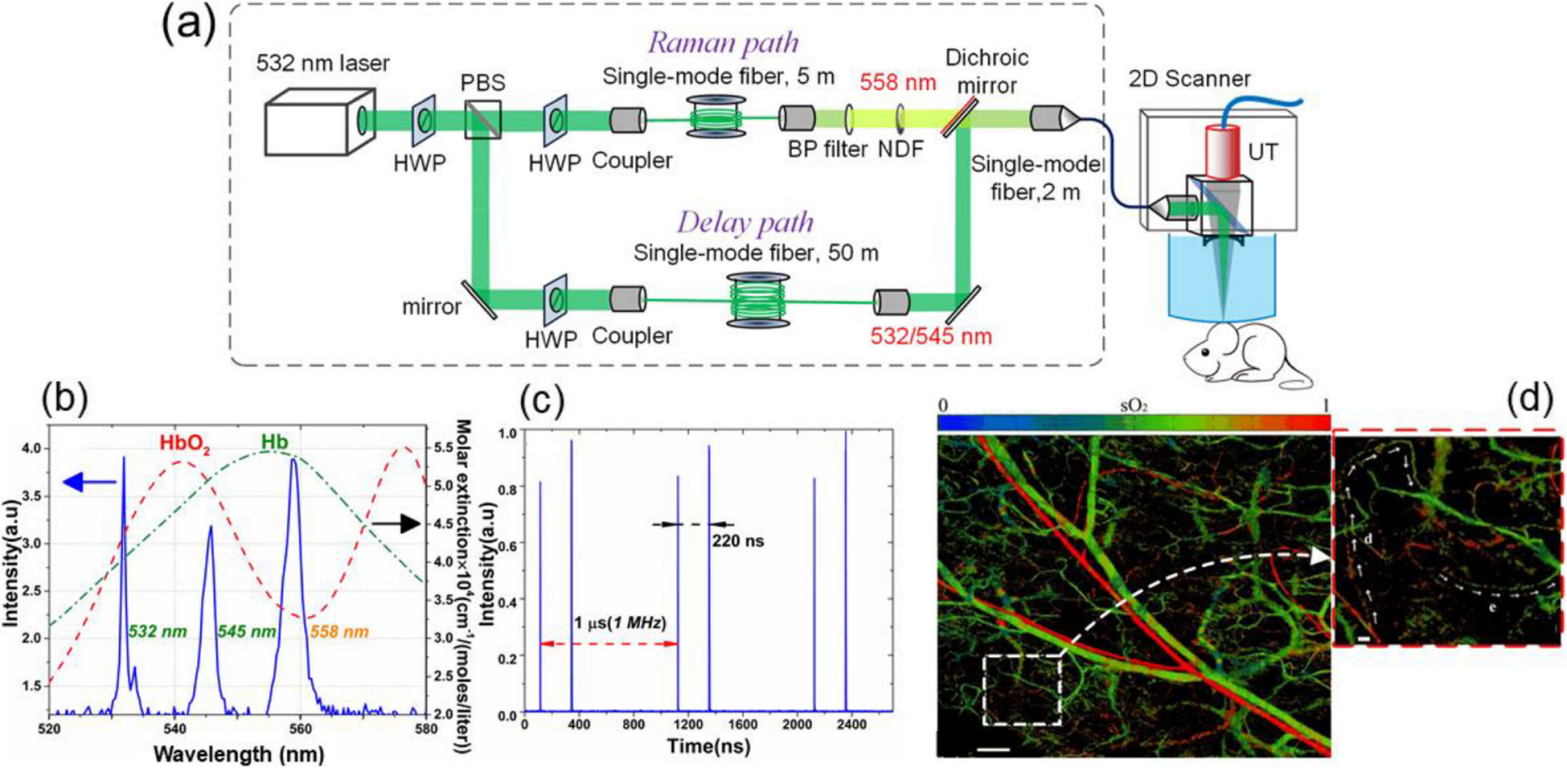
SRS-based multiwavelength fiber laser source for functional PAM. (**a**): Schematic of the laser and PAM; (**b**): Laser output spectrum; (**c**): Temporal signal of the output laser; and (**d**): In vivo oxygen saturation imaging result. Scale bar: 200 μm. Closeup shows the variation in oxygen saturation in single capillaries [34]

**Fig. 6:**
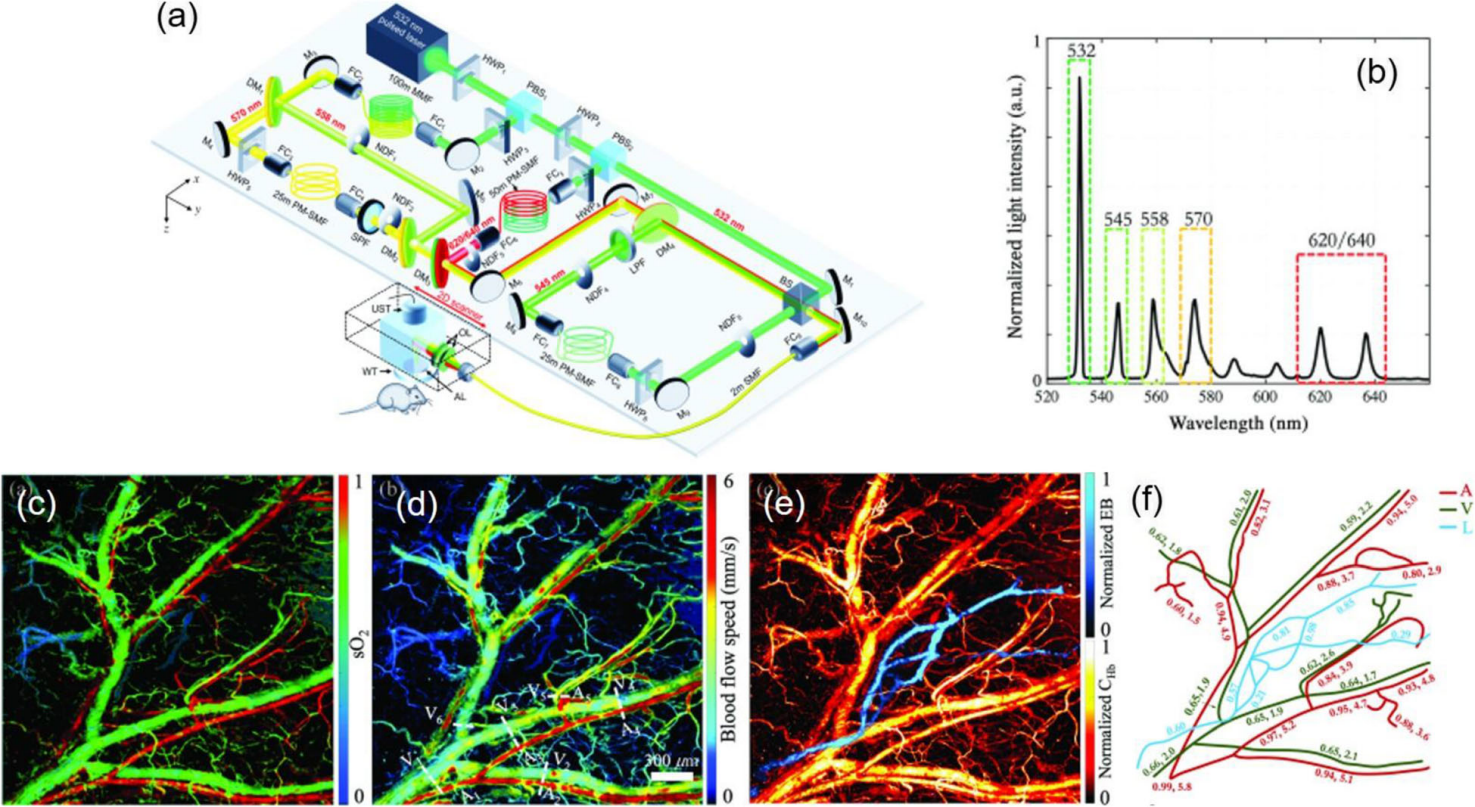
PAM with a five-wavelength SRS fiber laser source. (**a**): Schematic of the five-wavelength fiber laser and PAM; (**b**): Laser output spectrum; (**c**): Imaging result of oxygen saturation; (**d**): Blood flow speed determined using the dual-pulse method; (**e**): PAM imaging of the blood and lymphatic vessels; and (**f**): Variation of the oxygen saturation, blood flow speed, and relative lymphatic concentration from the root to the tip of a mouse ear. A: arteries; V: veins; L: lymphatic vessels [38]

Figure 5 shows an example of an SRS-based dual-wavelength fiber laser source in functional PAM [34]. The laser source has a 2-MHz repetition rate and a wavelength-switching time of 220 ns. In this source, the 532-nm pump laser was split into two beams, which were separately Raman-shifted to 558 nm (second-order Stokes wavelengths) and delayed by a low-nonlinearity optical fiber. The switching time is 220 ns, as determined by the optical path difference between the two beams. Such a short switching time can minimize the effect of a misalignment between the foci from the two excitation wavelengths. The two beams are then combined and focused on the biological tissue for scanning imaging. By using a spectral unmixing method, the hemoglobin concentration and oxygen saturation can be simultaneously imaged [17]. With an ultrashort wavelength switching time, PAM enables the functional imaging of individual flowing blood cells. This method was further extended to a triple-wavelength version (532/545/558 nm), by which the hemoglobin concentration, oxygen saturation, and blood flow can be measured and imaged at the same time [35–37]. Recently, the Raman fiber laser has extended to a five-wavelength output at 532, 545, 558, 570 and 620/640 nm, as shown in Fig. 6 [38]. With more laser wavelengths, the functionality of the PAM has been enhanced in that the hemoglobin concentration, oxygen saturation, blood flow, and dye-labeled lymphatic vessels can be simultaneously imaged.

SR S has enabled powerful light sources for PAM, which can output multiple wavelengths with a sufficient pulse energy for in vivo applications. In contrast to the optical gain offered by a rare-earth dopant, the Raman gain is not selective for the pump laser. As a result, the SRS-based source was also demonstrated with a 1060-nm pump laser, which can cover the lipid absorption band at 1210 nm [39]. Furthermore, by controlling the optical delays of each wavelength, functional imaging can be achieved using a single laser source. Notably, the SRS effect is sensitive to the polarization states of the pump and signal light. A polarization-maintaining fiber with an asymmetrical geometry or refractive index profile should be used for the Raman process. The maximum affordable power is approximately 100 μJ (depending on the mode area), which is limited by the damage threshold of the fiber.

To enhance the functionality of SRS-based sources for PAM, the following issues should be addressed: First, the silica fiber offers a 13-THz Raman shift (13-nm shift at 532 nm pump wavelength), which is insufficient to cover a wide spectral range. A larger wavelength shift will rely on the use of an optical fiber from a different host material, for example, soft glasses or gas-filled fibers. Second, because the Stokes wavelengths are fixed by the fiber material, precision control of the target output wavelength is a challenge. A weak pulse or CW seed laser exactly at the target wavelength can act as the signal light, which can be significantly amplified during a nonlinear process [40]. The amplification efficiency can also be enhanced because of suppressed spontaneous Raman scattering. A ‘real’ Raman laser scheme may also be taken into consideration. In such a system, a Raman laser cavity can be constructed using two wavelength-matched Bragg gratings for optical feedback at the target Raman-shifted wavelength. However, fiber Bragg gratings can only have reflective wavelengths at near-infrared wavelengths. Third, selectively enhancing one of the Stokes waves is difficult. The wavefront shaping technique has recently been applied to selectively strengthen or weaken one of the Stokes waves in multimode fibers by engineering the input wavefront of the pump laser [41]. This might open up a new possibility for the flexible control of SRS-based light sources.

Table 1 compares the output performance of the three types of fiber laser sources for PA excitation. Among the three types, the Q-switched laser has a higher output pulse energy and narrow spectral width, but the repetition rate is limited to tens of kilohertz. The supercontinuum source can cover a wide spectral range, which is beneficial for multispectral PAM. However, owing to the low spectral density of the output energy, optical filters with a spectral width of above 30 nm were used to obtain a sufficiently high pulse energy. The Raman laser can output multiwavelength, temporally separated pulsed trains, which are favorable for functional imaging, such as mapping the oxygen saturation. The repetition rate can be as high as 1 MHz, which can accelerate the imaging speed of PAM and visualize dynamic processes in vivo. Table 2 compares the performances of two commercial lasers that have been widely applied for PAM, i.e., a solid-state laser and a fiber laser. Both lasers emit green light at 532 nm. The fiber laser can have a higher pulse energy and tunable pulse width by using a master oscillator power amplifier (MOPA) scheme. In the MOPA scheme, a weak signal light is modulated using an intensity modulator before amplification over a rare-earth-doped fiber. Multiple high-power, low-coherence semiconductor lasers can be used for effective signal amplification. In addition, a double-cladding gain fiber with an asymmetrical cladding transverse geometry is typically applied, which allows the pump light in the inner cladding to pass through the core many times to maximize the output power [42, 43]. In MOPA lasers, individual parameters, including the pulse energy, duration, and repetition rate, can be flexibly adjusted, which can benefit its PAM application to maximize the strength of the photoacoustic signals.

**Table 1:** Performances of different fiber laser sources for photoacoustic excitation

Laser type	Output wavelength	Pulse energy	Repetition rate	Pulse width	Spectral width
Q-switched fiber laser [21]	1700, 1725 and 1750 nm, switchable	75.3 μJ at maximum (at 1750 nm)	10 kHz	16.7 ns	0.1 nm
Supercontinuum fiber laser [29]	1440–1850 nm, switchable	18.3 μJ	100 kHz	7 ns	33–38 nm
Raman-based fiber laser [38]	532, 545, 558, 570 and 620/640 nm, temporally separated	> 90 nJ	1 MHz	< 7 ns	3 nm

**Table 2:** Performances of a solid-state laser and a fiber laser for PAM

Laser product	Output wavelength	Pulse energy	Repetition rate	Pulse width	Stability
Elforlight SPOT-10-200-532	532 nm	20 μJ	0–50 kHz	1.8 ns	< 2%
Spectral Physics VGEN-G-HE-30	532 nm	180 μJ	Single pulse to 1500 kHz	5 to 30 ns, tunable	< 2%

## Fiber-laser-based ultrasound sensors

During the 1970s, a fiber-optic sensor was developed to detect underwater acoustic waves at audible frequencies for defense applications [44, 45]. An acoustically induced optical phase change can accumulate over the entire fiber length, yielding a high sensitivity. During the 1990s, the fabrication of a DFB laser in an optical fiber was made possible owing to the progress in multi-dopant optical fibers and fiber grating technology [12, 14, 46]. This laser cavity, typically several millimeters to centimeters in length, contains a phase-shifted Bragg grating or a grating pair that offers a strong optical feedback. A high-concentration rare-earth dopant can provide a sufficiently high gain with such a short cavity length. A DFB fiber laser was applied as a high-sensitivity sensor by measuring the lasing-frequency variation using interferometry, offering a high sensing resolution owing to the narrow linewidth and high signal-to-noise ratio of the laser output. Compared with existing interferometry-based hydrophones, fiber-laser-based acoustic sensors are advantageous because of their small size and intrinsic multiplexing capability.

Optical fibers are inherently sensitive to ultrasound waves at megahertz frequencies [47, 48]. The output phase change has a frequency- and polarization-dependent response. However, its application in ultrasound detection is significantly hindered because of its extremely short acoustic wavelength. In many cases, the response is hardly detectable owing to the limited interaction length between the optical fiber and ultrasound waves. To address this, a highly sensitive fiber-laser-based ultrasound sensor was developed and successfully applied during PAM [49]. In this section, the sensing mechanism, system configuration, sensor performance, and PAM applications of this new ultrasound sensor are reviewed.

### Response mechanism to ultrasound waves

**Fig. 7:**
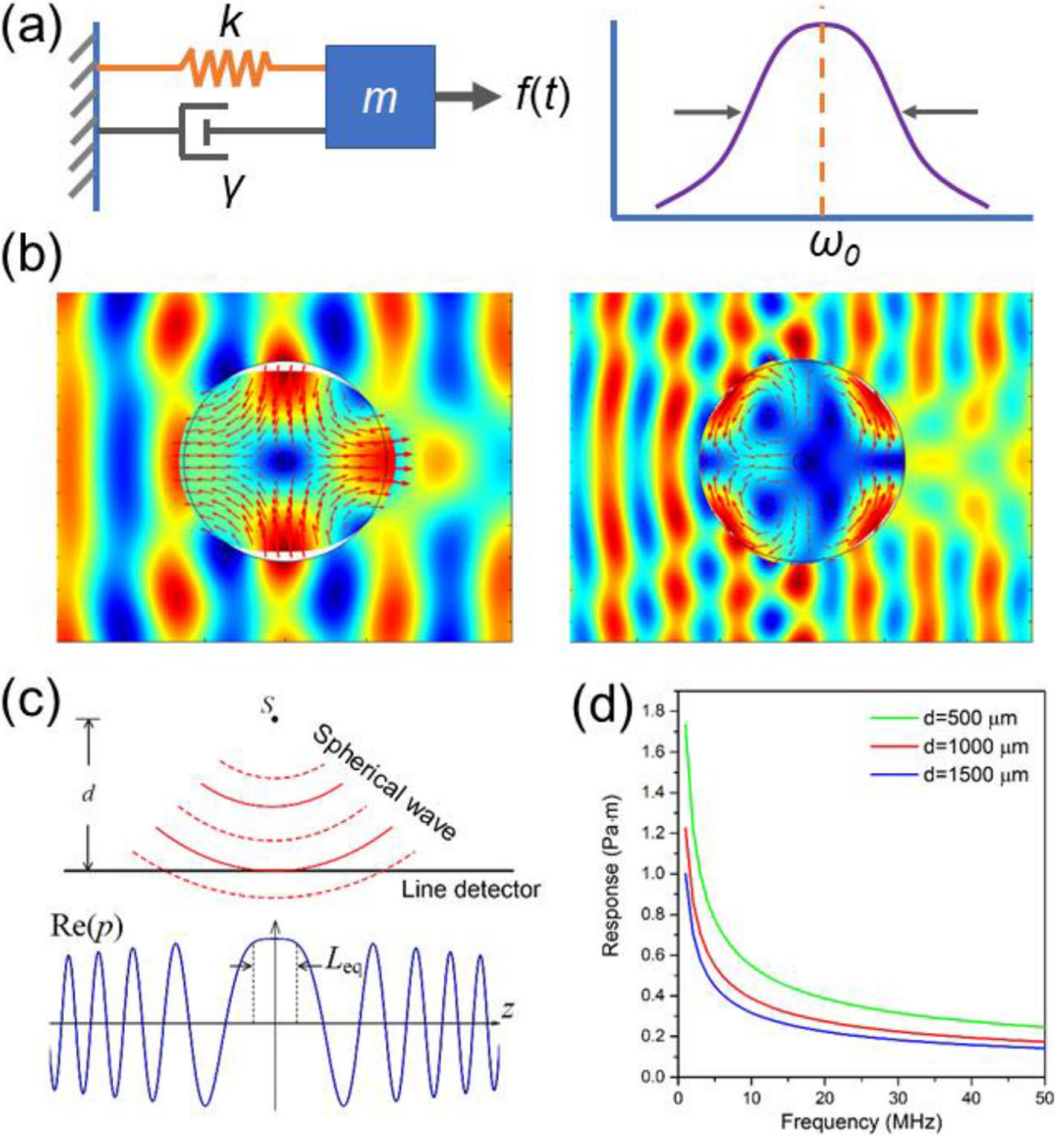
**a** Analogous model of a spring-mass oscillator. **b** Calculated deformation of the fiber surrounded by water in response to planar ultrasound waves. Left: (2,1) torsional-radial mode at 22 MHz. Right: (2, 2) torsional-radial mode at 39 MHz. SRS-based multiwavelength fiber laser source for functional PAM. (**a**): Schematic of the laser and PAM; (**b**): Laser output spectrum; (**c**): Schematic of the phase cancelation effect; and **d**: Effective interaction length with different source-to-fiber distances [50]

Prior to a specific description of the sensor fabrication and characterization, how the optical fiber, as an elastic cylinder, deforms in response to ultrasound waves is briefly described. This response is analogous to a dampened harmonic oscillator, for example, a mass-spring oscillator, as shown in Fig. 7(a). In this model, mass *m* is tied to a spring with Hook’s coefficient *k*. The vibration is driven by an external force *f*(*t*) and dampened with a coefficient *γ*. According to Newton’s second law, the frequency response has a Lorentz profile of 1/[i(*ω*_0_-*ω*) + *ω*_0_*γ*]. The induced vibration can be characterized by a central frequency of *ω*_0_ = (*k*/*m*)^1/2^ and quality factor of *Q* = (*m·k*)^1/2^/ *γ*. Now, considering the vibration of an optical fiber immersed in water, it is assumed that the wave vector of the incident ultrasound is orthogonal to the fiber, and that a two-dimensional model is applicable. Analogous to the mass-spring model, the vibration frequency is determined based on the elastic and geometric parameters of the fiber, including the density, acoustic velocities, and fiber diameter. Its vibration is driven by the ultrasound applied and dampened by the surrounding water. The acoustically induced response can be considered as a weak mechanical resonance of the fiber, which is dampened by the acoustic interaction between the cylinder and water. The differences between the cylinder vibration and the spring-mass oscillator are as follows: First, the cylinder vibrates in different modes, which can be denoted by an azimuthal order *l* and radial order *n*. Among all vibration modes of an optical fiber, the axially symmetrical modes (with *l* = 0) and torsional-radial modes (with *l* = 2) can induce changes in the optical phase. Figure 7(b) shows the first and second order *l* = 2 modes of an optical fiber excited by acoustic waves. Second, the frequency response of the cylinder vibration is expressed by Bessel functions of *k*_a_*r*_t_, where *k*_a_ and *r*_t_ denote the acoustic wavenumber and radial position, respectively. Therefore, this response deviates somewhat from the well-known Lorentz curve. The exact calculation of the ultrasound response depends on the use of an acoustic scattering model by solving multiple linear equations. The incident wave is decomposed as a linear summation of cylindrical waves as input signals, and the induced vibrational amplitude and phase of the individual vibration modes can be calculated [48, 51].

It is worth noting that PAM detects spherical ultrasound waves rather than planar waves. Here, the acoustic response of an ideal line detector to a spherical wave is discussed. As shown in Fig. 7(c), point source *S* generates an acoustic wave with frequency *f*_a_. Suppose that the acoustic wave is completely absorbed by the line detector placed at a distance *d*. The acoustic pressure with propagation can be written as *p*(*ω, r*) = exp(−i*k*_a_*r*)/*r*, where *r* = (*d*^2^ + *z*^2^)^1/2^ is the propagation distance, and *z* represents the longitudinal position (a stricter expression can be found in ref. [21]). The spherical wavefront arrives at the line detector in different phases. The response of the line detector can be expressed as integration $R=\underset{-\infty }{\overset{+\infty }{\int }}\frac{\mathit{\exp}\left(-i{k}_ar\right)}{r} dr$ and simplified as $\frac{2.506}{\sqrt{k_ad}\ }$. Figure 7(d) plots the frequency response, showing that a higher acoustic frequency leads to reduced sensitivity, following a 1/*f*_a_^1/2^ variation. This is a result of the faster acoustic-phase oscillation along the line detector and the stronger self-cancellation effect. The response can be equivalently considered as an effect of a uniform pressure *p*_eq_ = 1/*d* (the amplitude at the position facing the source *z* = 0) with a length *L*_eq_. Based on the above relation, the equivalent interaction length can be written as ${L}_{eq}=2.506\sqrt{d/{k}_a}$, which is 10- to 100-times shorter than the sensitive region (typically 2–10 mm) of a fiber laser sensor. For a 50-MHz spherical ultrasound wave (wavelength of 30 μm, and speed in water of 1500 m/s) with a source-detector distance of *d* = 500 μm, the equivalent interaction length was *L*_eq_ = 122 μm [50].

Based on the above analysis, it was found that a conventional interferometric sensor, which relies on a sensitivity proportional to the fiber length, is unsuitable for detecting ultrasound waves with sub-millimeter wavelengths. To achieve a high SNR, the overlap between the ultrasonic interaction region and the optical sensor should be enhanced. In recent years, ultrasound sensors based on optical resonance have been developed, including planar and fiber-based Fabry–Perot sensors, for application to in-fiber phase-shifted gratings, polymer microring resonators, and silicon-on-insulator nano-resonators [52–56]. These sensors offer a high sensitivity and a wide detection bandwidth. However, the stable sensor output relies on a frequency locking of the probe light at the quadrature point. This feedback-based locking becomes extremely difficult for a resonator with a high quality factor (or *Q* factor), which hinders its application in PAM. A DFB laser can be equivalently viewed as an active resonator, in which the cavity offers a gain instead of a loss, and is a candidate for ultrasound detection for the following reasons: (1) The laser output has a 1/*f* noise spectrum, and the noise level at megahertz frequencies is extremely low. (2) The measurement of the acoustically induced lasing-frequency change does not necessarily require frequency-locking feedback. Heterodyning fiber lasers, which have a dual-polarization-mode output, can output radio-frequency beat notes, whose frequency can be measured using a commercial photodetector. The capability of the sensor to detect ultrasound waves for PAM use is described in the following text.

### Sensor performance and PAM results

The laser sensor has a cavity length of less than 8 mm, emits two orthogonal polarization modes at the communication band, and outputs a beat signal at approximately 2 GHz. Ultrasound waves can change the fiber birefringence and induce a detectable change in the beat frequency. The sensor performance is briefly described in ref. [57]:

#### Bandwidth

**Fig. 8:**
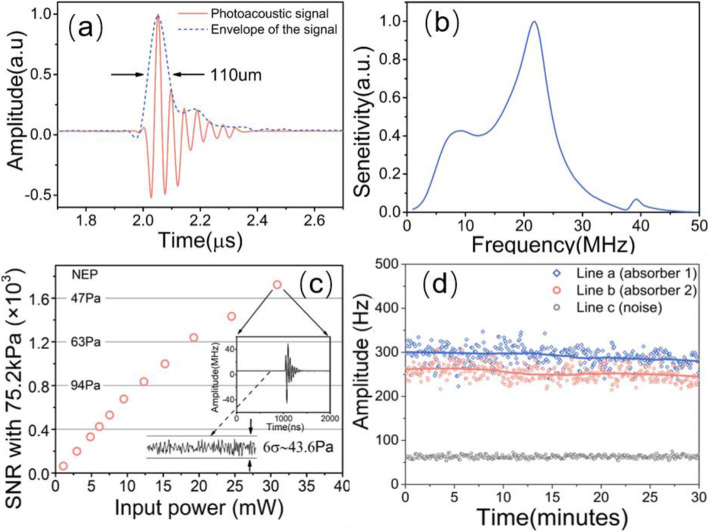
**a** and **b**: Temporal (**a**) and frequency (**b**) response of the sensor; (**c**) Measured noise equivalent pressure as a function of input power at the photodetector; and (**d**) Stability test result by continuously measuring the photoacoustic signals from two discrete absorbers for over 30 min [57].

Figures 8 (a) and (b) show the temporal waveform and the corresponding spectrum of the same PA signal. The central frequency of this mechanical mode is approximately 22 MHz, and the − 6 dB bandwidth is approximately 70%. This provides a 110-μm axial resolution. The central frequency depends on the diameter of the fiber (approximately 125 μm). The working bandwidth can be extended by etching the fiber with hydrofluoric acid. For example, a 65-μm sensor provides a twofold bandwidth extension, and the central frequency shifts to approximately 40 MHz.

#### SNR

Figure 8 (c) shows the measured SNR at different fiber laser powers with a fixed peak-to-peak acoustic pressure of an incident ultrasound wave at 75.2 kPa. The SNR is mainly limited by the noise of the photodetector. In the high input power regime, the dominant noise source is shot noise, and the SNR is approximately proportional to the square root of the optical power. To achieve a high signal-to-noise ratio, the incident optical power must be increased as much as possible, and the photodetector must operate within the shot noise-limited region. Eventually, the saturation of the photodetector limits the optical power. When the input power on the photodiode is 30 mW, the noise-equivalent pressure is approximately 43.6 Pa with a 50-MHz acquisition bandwidth.

#### Stability

Figure 8 (d) shows the stability test result of the sensor by measuring the photoacoustic signals from two discrete absorbers excited using a linearly scanning pulsed laser. The 30-min measurement result showed no noticeable variation in sensitivity. It was also observed that even with changes in vibration or temperature, the sensor remained stable with a high sensitivity.

**Fig. 9:**
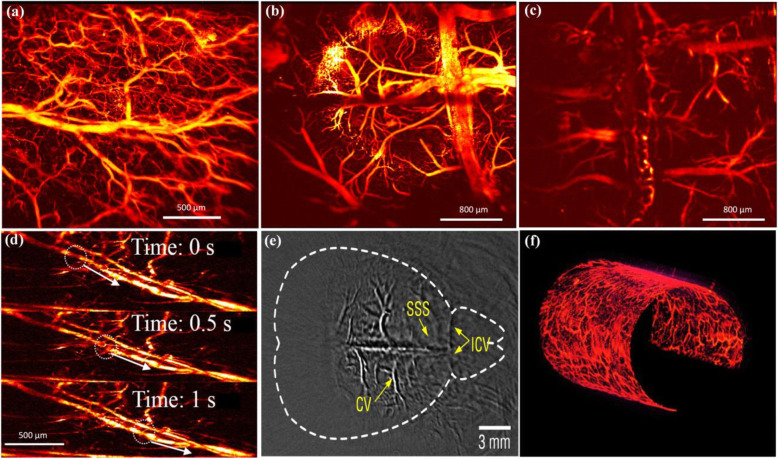
In vivo PAI using the fiber-laser ultrasound sensor. (**a**): Fast-scanning optical-resolution PAM of mouse ear; (**b**) and (**c**): In vivo imaging of the mouse brain (**b**) without and (**c**) with intact skull; (**d**): Three consecutive snapshots showing the negative contrast flow in a blood vessel; (**e**): In vivo photoacoustic computed tomography of a mouse brain; (**f**): In vivo endoscope image of a rat rectum [58, 59]

Figures 9(a)–(d) show the in vivo PAM images of a mouse ear and brain, using a fiber laser sensor for ultrasound detection [58, 59]. During PAM, the focused 532-nm nanosecond laser beam raster scans as the sensor is mounted horizontally at 1.6 mm above the sample. Figure 9(a) shows a vessel image of the mouse ear. The laser pulse repetition rate is 100 kHz. The B-scan rate is 100 Hz, and the frame rate for a volumetric image is 0.2 Hz. The image has a pixel resolution of 500 × 500, and the field of view is 2 × 2 mm^2^. The trunk vessels and capillaries can be resolved. Figures 9(b) and (c) show the in vivo PAM results of a mouse brain under the same conditions, with and without an intact skull. The fiber-optic sensor can offer sufficient sensitivity even with an intact skull, although the capillaries become less invisible as a result of optical scattering. Figure 9(d) shows the blood flow dynamics obtained by increasing the frame rate to 2 Hz. In this case, the laser repetition rate was 160 kHz, and the B-scan rate was 400 Hz. Each volumetric image had 200 × 200 A-lines. The step size in the lateral direction was 10 μm. Figure 9(e) shows the photoacoustic computed tomography results of a mouse brain obtained by rotary scanning the sensor [59]. Figure 9(f) shows the photoacoustic endoscopy results of a mouse rectum, taking advantage of the small size of the sensor. In this case, the fiber laser sensor can provide a high sensitivity without the need for acoustic focusing, making the catheter small and compact.

## Discussion and conclusion

The development of fiber laser technology is still in progress in terms of both output performance and market scale. It is possible for most PAMs to adopt cost-effective fiber laser sources. In addition, different nonlinear wavelength-shift processes can be utilized for wavelength extension in multispectral PAM. Researchers are investigating how to achieve a satisfactory output performance at all wavelengths of interest. At the sensor end, the DFB laser offers a single-frequency, low-noise sensing element for ultrasound detection. The fiber-laser-based sensor has a small size and is highly sensitive. Because the sensing signal is located at radio frequencies, new techniques based on microwave photonics can be adopted to further enhance the SNR. In addition, the output wavelength and beat frequency can be controlled for each sensor, offering the possibility of multiplexing. A wavelength/time division multiplexing technique will also be realized to form a sensor array that is interrogated using a single demodulation system.

In contrast to conventional PAM instruments, fiber-laser-based counterparts can have a significantly reduced weight. As a result, they can be applied in handheld and head-mounted imaging modalities, including a head-mounted PAM for monitoring the brain activities of a small animal weighing more than 20 g that has difficulty being mounted for a long period of time. The weight of a PAM instrument can be significantly reduced by using fiber lasers as light sources and sensors. In addition, optical-fiber-based endoscopy has been successfully used in diagnostic applications. The history of these endoscopic imagers dates back to 60 years ago, even before the proposal and demonstration of a low-loss communication fiber. However, the functionality of such endoscopes is limited to an image of the surface structure of an organ. Fiber lasers can enable an all-fiber PA endoscope, which can be used for the detection of cancer at an early stage.

## Data Availability

The data that support the findings of this study are available from the authors upon reasonable request.

## Abbreviations

PAM: Photoacoustic microscopy
SRS: Stimulated Raman scattering
PAI: Photoacoustic imaging
MOPA: Master oscillator power amplifier
PCF: Photonic crystal fiber
ZDW: Zero-dispersion wavelength
DFB: Distributed feedback
UV: Ultraviolet
OCT: Optical coherent tomography
